# A Neurophysiological Event of Arousal Modulation May Underlie fMRI-EEG Correlations

**DOI:** 10.3389/fnins.2019.00823

**Published:** 2019-08-07

**Authors:** Feng Han, Yameng Gu, Xiao Liu

**Affiliations:** ^1^Department of Biomedical Engineering, The Pennsylvania State University, State College, PA, United States; ^2^Institute for CyberScience, The Pennsylvania State University, State College, PA, United States

**Keywords:** fMRI and EEG correlation, resting-state connectivity, transient arousal modulation, global signal, electrophysiological signal

Correlation between functional Magnetic Resonance Imaging (fMRI) and electroencephalogram (EEG) concurrently recorded at rest has been studied extensively with the purpose of locating sources of EEG alpha waves (8–12 Hz) in earlier years (Goldman et al., 2002; Moosmann et al., 2003; Feige et al., 2005) but for understanding the neurophysiological correlates of resting-state fMRI (rsfMRI) signals more recently (Mantini et al., 2007; Liu et al., 2012). In this opinion paper, we will first review two lines of research on rsfMRI-EEG correlations and neural correlates of the global rsfMRI signal. We will then present our major hypothesis regarding the role of an arousal event in the fMRI-EEG correlation based on evidence for these studies. Lastly, we will discuss the potential impacts of future research on the validation of our hypothesis or similar topics.

Despite some minor deviations (Laufs et al., 2003), the EEG alpha-band power was consistently found to have strong, widespread negative correlations with fMRI signals at the sensory/motor areas but positive correlations with more circumscribed regions at the anterior and medial parts of the thalamus (Goldman et al., 2002; Moosmann et al., 2003; Feige et al., 2005; Liu et al., 2012). A similar spatial pattern of correlations was also found between rsfMRI and EEG vigilance index (Falahpour et al., 2018) that is defined as the ratio of alpha to delta-theta (1–7 Hz) powers, suggesting the observed fMRI-EEG correlations are likely related to vigilance fluctuation. Other than the direct correlations, the cross-modality fMRI-EEG relationship is also indicated by the dependency of rsfMRI connectivity on bandlimited EEG powers. At the subject level, the global rsfMRI signal, i.e., the whole-brain average, and spatially non-specific connectivity of individuals are negatively correlated with their EEG vigilance index (Wong et al., 2013). On a finer timescale, dynamic resting-state connectivity (over time windows of 1–2 min) between the default mode network (DMN) and dorsal attentional network (DAN) was found dependent on the EEG alpha power, with stronger anti-correlation (corresponding to a smaller global signal) appearing with higher EEG alpha power (Tagliazucchi et al., 2012; Chang et al., 2013). Thus, the global rsfMRI signal, as well as spatially non-specific connectivity it induces, appears to be stronger for individuals (or time windows) with lower EEG alpha power and/or vigilance. These findings are consistent with a series of studies showing that the global rsfMRI signal and/or non-specific rsfMRI connectivity is stronger at light sleep stages (Fukunaga et al., 2006), after sleep deprivation (Yeo et al., 2015), or after taking hypnotic drugs (Kiviniemi et al., 2005; Licata et al., 2013), but can be effectively reduced with caffeine administration (Wong et al., 2013). Therefore, the key to the puzzle lies in understanding the neural basis of the global rsfMRI signal.

The search for the neural basis of the global rsfMRI signal has recently made significant progress with the help of intra-cranial electrophysiological recordings from monkeys. A stereotypical event with a characteristic time-frequency pattern was identified in the global signal of large-scale electrocorticography (ECoG) recordings from monkeys (Liu et al., 2015). This sequential spectral transition (SST) event lasting 10–20 s starts with an abrupt reduction in the middle-frequency alpha-beta (8–30 Hz) activity, which is followed by an increase of the low-frequency delta-theta power (< 8 Hz) and a burst of high-frequency broadband gamma activity (>30 Hz) (Figure 1A). The middle-to-low frequency power transition at the SST event may indicate a transient modulation in brain arousal level (Steriade, 2006; Harris and Thiele, 2011). Consistent with this notion, the SST events are strong and occur most frequently during light sleep and drowsy states, much less under a sleep-conducive eyes-closed condition, and almost absent under a more alert eyes-open condition (Liu et al., 2015). This brain state dependency is very similar to the global rsfMRI component (Fukunaga et al., 2006; Jao et al., 2013; Wong et al., 2013), suggesting a close relationship between the two. With concurrent fMRI-electrophysiology recordings from another group of monkeys, the SST event was found to be indeed one-to-one coupled with widespread rsfMRI changes that are shown as a single large peak in the global rsfMRI signal (Liu et al., 2018) (Figures 1B,C). Two other observations further support the correspondence between the SST event and global rsfMRI peak. First, the global rsfMRI peak shows larger signal increases at the sensory/motor regions, including the auditory, somatosensory, motor, and visual cortices (Liu et al., 2018), and this sensory-dominant pattern is similar to the spatial profile of gamma-power increase at SST (Liu et al., 2015). Secondly, the widespread fMRI signal increases at the global peaks are actually associated with fMRI signal decreases at very specific subcortical regions, including the nucleus basalis of the basal forebrain and the dorsal midline thalamus (DMT) known to be involved in arousal regulation (Liu et al., 2018). This finding is consistent with the middle-to-low frequency transition at the SST indicative of arousal modulations. These studies provide neurophysiological understanding not only for the global rsfMRI signal but also for its close relationship with brain arousal states (Kiviniemi et al., 2005; Fukunaga et al., 2006; Horovitz et al., 2009; Tagliazucchi and Laufs, 2014; Yeo et al., 2015).

**Figure 1:**
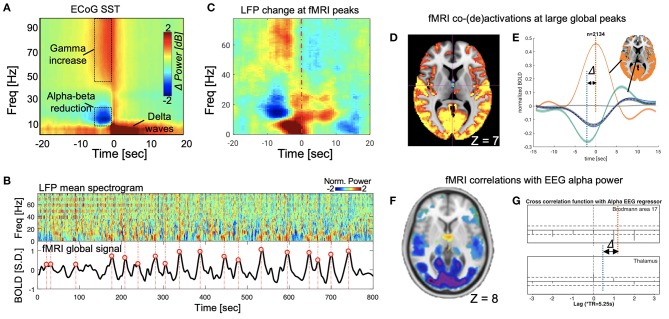
SST events and its parallelism with global fMRI peaks in monkeys, and the correspondence between the global fMRI co-activations and fMRI-EEG correlations in human. **(A)** The SST event observed from monkey ECoG data (Liu et al., 2015). **(B)** The correspondence between the SST events (upper panel) in the local field potentials (LFPs) mean spectrogram and the large fMRI global signal peaks (lower panel) (Liu et al., 2018). **(C)** The SST pattern emerges when averaging the mean LFP spectrogram segments at large global fMRI peaks (locations aligning with red circles in **B**) (Liu et al., 2018). **(D)** Significant fMRI co-activations (orange) and de-activations (cyan) at the large global peaks (averaged over 2,134 peaks in total), which have been shown to be coupled with the SST events in electrophysiological data (Liu et al., 2018). The axial slice is shown at *Z* = 7 mm in the MNI space. **(E)** The fMRI de-activations at the dorsal midline thalamus actually reach its peak (negative) 2–3 s before that of the fMRI co-activations at widespread cortical regions, particularly the sensory/motor cortex. **(F)** The fMRI correlations to the alpha-band EEG power show strong negative correlations at the sensory/motor regions but positive correlations at the midline thalamus. The slice is shown at *Z* = 8 mm in the MNI space (Liu et al., 2012). **(G)** The cross-correlations between the fMRI and EEG signals as a function of their time lags. The peak negative correlation (orange dash line, approximated according to values at lags) at the visual cortex, i.e., the Brodmann area 17, and the peak positive correlation (cyan dash line) at the thalamus are found at different time lags, suggesting the fMRI changes at these regions are delayed by a few seconds (Feige et al., 2005).

Combining evidence from these two lines of research, we propose our hypothesis that the fMRI-EEG correlations observed in human literature results mainly from transient arousal modulations manifested as SST events. We formulated this hypothesis based on the following evidence. First, the power modulations at SST events and associated fMRI signal changes are much larger than those in other time periods (Liu et al., 2015) and the fMRI-EEG correlation they cause is thus expected to be stronger than other sources of arousal irrelevance. Secondly, the fMRI-EEG correlation shows a spatial pattern consistent with those of SST-related modulations. The fMRI correlations to the EEG alpha power (or EEG vigilance index) (Feige et al., 2005; Liu et al., 2012; Falahpour et al., 2018) display a sensory-dominant pattern that resembles both the spatial profile of the SST gamma-power increase (Liu et al., 2015) and the fMRI co-activation pattern at the large global peaks (Liu et al., 2018). The negative correlation is expected given that the reduction of alpha power at the SST is associated with sensory-dominant fMRI signal increases. More importantly, the widespread negative fMRI-EEG correlations are accompanied by focal positive correlations at the DMT. Correspondingly, the fMRI co-activations at the global peaks are associated with opposite changes, i.e., de-activations, at exactly the same thalamic region (Figures 1D,F) (Liu et al., 2012, 2018). Thirdly, the fMRI-EEG correlations also show similar temporal dynamics as the SST-associated fMRI changes. The positive fMRI-EEG correlations found at the thalamus reach their peak with a delay of ~2.5 s between the two modalities, which is 2–3 s shorter than the typical hemodynamic delay that was found true for the negative fMRI-EEG correlations at the sensory/motor regions. Close inspection of region-specific fMRI dynamics at the large global peaks revealed a similar pattern of temporal delays: fMRI deactivation at DMT precedes the widespread fMRI co-activations, particularly those at the sensory/motor regions, by 2–3 s (Figures 1E,G) (Feige et al., 2005; Liu et al., 2018). Last but not least, the dependency of the fMRI-EEG correlations on brain arousal state can be well-explained by the SST events of transient arousal modulations. The fMRI-EEG correlations become significantly weaker not only from the sleep-conducive eyes-closed condition to alert eyes-open condition but also after caffeine administration (Falahpour et al., 2018). The occurrence of SST events during drowsy states could be the underlying reason for this state dependency.

The validation of this hypothesis and future research on this topic may have potential implications for the following aspects. First, the validation of this hypothesis will provide a more mechanistic understanding of empirical observation of fMRI-EEG correlations. It is worth noting that the validation of this hypothesis with simultaneous fMRI-EEG in human may face additional challenges, including the insensitivity of scalp EEG to gamma-band activity and the difficulty in controlling brain arousal state. Therefore, algorithms for detecting SST with frequency features below 30 Hz and procedures for promoting sleepiness of subjects could be important strategies for overcoming these obstacles. Moreover, the EEG source localization (Liu et al., 2017) may potentially enable a spatial comparison of two modalities at SST events and further confirm their correspondence spatially. Secondly, the validation of the hypothesis and related research may also advance our understanding of rsfMRI connectivity and its dependency on EEG activity. The widespread fMRI increase associated with SST events could result in widespread increases of rsfMRI connectivity, which could be larger for sensory/motor networks due to its sensory-dominant pattern. The thalamocortical connectivity can be modulated in a complex way given the opposite fMRI changes in cortical and thalamic regions seen at the global peaks, and the use of controversial global signal regression step would further complicate the scenario (Fox et al., 2009; Murphy et al., 2009; Gotts et al., 2013). Despite these complications, the SST-associated fMRI changes would likely introduce strong modulations in rsfMRI connectivity and associate them with EEG changes. This could be the reason for the dependency of non-specific rsfMRI connectivity on band-limited EEG power, either at the subject level (Wong et al., 2013) or across time windows of 1–2 min (Chang et al., 2013) that are much longer than the SST time span of 10–20 s. The introduction of correlation-based connectivity measures significantly complicates the fMRI-EEG relationship, and the key to solving this problem may be to focus on temporal events driving all these correlations (Liu and Duyn, 2013; Liu et al., 2013; Matsui et al., 2019), such as the SST event. Thirdly, the involvement of transient arousal events in the fMRI-EEG correlation may also provide a new perspective for understanding rsfMRI correlations with other non-neuronal signals, which has been a hot topic of rsfMRI research (Chang et al., 2016b). Strong correlations have been reported between rsfMRI signals and other measurements, such as cardiac (Shmueli et al., 2007) and respiratory (Birn et al., 2006) signals and head motions (Power et al., 2012; Van Dijk et al., 2012), which have been interpreted as non-neuronal contributions to rsfMRI. However, given the known links between arousal modulations and physiological changes (Luft and Bhattacharya, 2015; Penzel et al., 2016) and head motions (Van den Berg, 2006), we ought to re-think whether the rsfMRI correlations with the non-neuronal noise may also arise from transient arousal modulations, such as the SST event. This notion is consistent with a recent finding that the presence of physio-rsfMRI correlation is actually dependent on the EEG alpha power (Yuan et al., 2013). Additionally, a clear understanding of SST-associated fMRI changes may help to improve fMRI-based arousal measure, which is expected to have many potential applications. A template-matching algorithm has been proposed to measure brain arousal by comparing instantaneous fMRI co-activations with the fMRI correlation map of EEG vigilance, which shows a sensory-dominant cortical pattern and anti-phase change at the thalamus (Chang et al., 2016a; Falahpour et al., 2018). This fMRI-based arousal measure could be further improved with a detailed understanding of the spatiotemporal dynamics of SST-associated fMRI changes. An accurate estimation of brain arousal with rsfMRI is expected to have many potential applications, especially given the availability of rsfMRI data. For example, many psychiatric and neurodegenerative diseases, including Alzheimer's disease and Parkinson's disease, are known to concur with disrupted sleep and circadian rhythms (Wulff et al., 2010), and the widely available rsfMRI in these patient populations would immediately enable studies of arousal state changes in these diseases.

In summary, we argued, based on existing evidence, that a recently discovered neurophysiological event of arousal modulation may account for a significant portion of fMRI-EEG relationship, shown both as their direct correlations and the dependency of rsfMRI connectivity on EEG powers. Future studies should seek to test the validity of this hypothesis, which is important for understanding the neural origin of fMRI-EEG correlation, for interpreting rsfMRI connectivity and its dependency on EEG, for understanding rsfMRI correlations with other non-neuronal signals, and also for improving the fMRI-based arousal measure.

## Author Contributions

XL, FH, and YG contributed to the data acquisition and image processing. FH and XL made substantial contributions to the conception and design of the work. All authors contributed to writing the paper.

### Conflict of Interest Statement

The authors declare that the research was conducted in the absence of any commercial or financial relationships that could be construed as a potential conflict of interest.
